# Iceland as Stepping Stone for Spread of Highly Pathogenic Avian Influenza Virus between Europe and North America

**DOI:** 10.3201/eid2812.221086

**Published:** 2022-12

**Authors:** Anne Günther, Oliver Krone, Vilhjalmur Svansson, Anne Pohlmann, Jacqueline King, Gunnar Thor Hallgrimsson, Kristinn Haukur Skarphéðinsson, Heiða Sigurðardóttir, Stefán Ragnar Jónsson, Martin Beer, Brigitte Brugger, Timm Harder

**Affiliations:** Friedrich-Loeffler-Institute, Greifswald–Insel Riems, Germany (A. Günther, A. Pohlmann, J. King, M. Beer, T. Harder);; Leibniz Institute for Zoo and Wildlife Research, Berlin, Germany (O. Krone);; University of Iceland, Reykjavik, Iceland (V. Svansson, G.T. Hallgrimsson, H. Sigurðardóttir, S.R. Jónsson);; Icelandic Institute of Natural History, Garðabær, Iceland (K.H. Skarphéðinsson);; Icelandic Food and Veterinary Authority, Selfoss, Iceland (B. Brugger)

**Keywords:** highly pathogenic avian influenza virus, influenza virus, viruses, highly pathogenic avian influenza, avian influenza, HPAIV, H5N1, subtype, respiratory infections, North Atlantic, transatlantic spread, migratory birds, stepping stone, zoonoses, Europe, North America, Iceland

## Abstract

Highly pathogenic avian influenza viruses (HPAIVs) of hemagglutinin type H5 and clade 2.3.4.4b have widely spread within the northern hemisphere since 2020 and threaten wild bird populations, as well as poultry production. We present phylogeographic evidence that Iceland has been used as a stepping stone for HPAIV translocation from northern Europe to North America by infected but mobile wild birds. At least 2 independent incursions of HPAIV H5N1 clade 2.3.4.4b assigned to 2 hemagglutinin clusters, B1 and B2, are documented for summer‒autumn 2021 and spring 2022. Spread of HPAIV H5N1 to and among colony-breeding pelagic avian species in Iceland is ongoing. Potentially devastating effects (i.e., local losses >25%) on these species caused by extended HPAIV circulation in space and time are being observed at several affected breeding sites throughout the North Atlantic.

Potentially zoonotic highly pathogenic avian influenza (HPAI) viruses (HPAIVs) of subtype hemagglutinin (HA) 5 (H5) emerged from a domestic geese flock in southern China in the mid-1990s. Since then, descendants of this so-called goose/Guangdong (gs/GD) lineage have continued to circulate, evolved into various clades, and formed a plethora of subgenotypes and genotypes that threaten poultry production worldwide (*1*,*2*). Because of repeated incursions from poultry into migratory aquatic wild bird populations in Asia, these viruses have spread, since 2005, in several waves westward and southward across Eurasia, into Africa and eastward, through the Bering strait, into North America. Infected but mobile migratory birds aided in linking geographically widely separated areas along overlapping flyways; palearctic breeding areas were serving as an additional link between Eurasia and America during 2014 (*3*,*4*).

Because Europe was facing the most severe HPAIV epizootics in the influenza winter seasons of 2020–21 and 2021–22 in terms of case numbers and genetic diversity of characterized viruses (*5*,*6*), concerns about spread to North America, this time by westward virus spread, were renewed. By December 2021, HPAI H5N1 detection in wild birds in Canada was reported, followed by numerous additional wild bird cases and incursions into poultry holdings along the eastern coastline of the United States (*7*,*8*). Phylogenetic analyses of the viruses in North America confirmed a close relationship to HPAIV H5N1 genotypes from Europe (*7**–**9*). Although the outcomes of the transatlantic HPAIV transfer are evident, the steps taken by the virus to cross the Atlantic are not. We present data supporting HPAIV transfer from Europe to North America by bird migration through Iceland.

## Incursion of HPAIV H5N1 into Iceland

Although low pathogenicity avian influenza virus (AIV) strains have been detected in sea birds around Iceland (*10*,*11*), outbreaks of HPAIV were not reported from Iceland until spring 2022. However, retrospective screening of wild bird samples from Iceland showed that an HPAI case was in a juvenile white-tailed sea eagle (*Haliaeetus albicilla*) found dead in the southern Westfjords, Iceland, during October 2021 (*12*). This bird had been equipped with a satellite transmitter (global positioning system/global system for mobile communications) as a nestling on July 24, 2021. After fledging on August 11, 2021, the eagle stayed in the nesting area of its parents and moved within a range of 1.6 km^2^ (95% minimum complex polygon) for ≈2 months. The juvenile eagle died at the shore of the region in Iceland on October 8, 2021, and was kept frozen until necropsy in the spring of 2022.

Postmortem examination showed a female weighing 5,540 g that had extensive subcutaneous and body cavity fat tissue indicating a good nutritional condition. Gross pathologic alterations (fibrinous pericarditis, swollen hyperemic liver, spleen, and kidneys) were indicative of a severe infectious disease, which led to an acute death of the young eagle. We analyzed organ samples for AIV by using quantitative reverse transcription PCR as described (*13*). HPAIV of subtype H5N1 was found at high viral loads in all tissue samples examined, including the brain (cycle threshold 16.2).

Despite an appeal from the veterinary authorities in Iceland to the general public to report finding of sick or dead wild birds, only 17 birds came to be sampled and AIV was tested in the first 9 months of 2021, and all samples were AIV negative. In the beginning of 2022, the veterinary authorities in Iceland enhanced passive surveillance through reports from the public of sick or dead wild birds. In mid-April, a common raven (*Corvus corax*) and a pink-footed goose (*Anser brachyrhynchus*) tested HPAIV H5N1 positive. In addition, in the same period, a northern gannet (*Morus bassanus*) tested positive for H5N1, but HPAI could not be confirmed. The raven was found on a farm in southern Iceland where 6 days later a backyard chicken flock on the same farm showed abruptly increased mortality rate, and chicken carcasses tested HPAIV H5N1 positive. Consequently, public awareness and reporting of dead wild birds increased markedly after a press release on these first findings.

From April 2022 onward, including the already identified wild birds, HPAIV H5N1 was detected in 21 wild birds from 10 species: northern gannets (n = 7), European herring gull (*Larus argentatus*) (n = 2), great black-backed gull (*Larus marinus*) (n = 2), great skua (*Stercorarius skua*) (n = 2), greylag goose (*Anser anser*) (n = 2), pink-footed goose (n = 2), barnacle goose (*Branta leucopsis*) (n = 1), black-headed gull (*Chroicocephalus ridibundus*) (n = 1), common raven (n = 1), and lesser black-backed gull (*Larus fuscus*) (n = 1). Because in 1 sample from a northern gannet, neuraminidase 1 could not be confirmed, the bird was reported as positive for HPAIV H5Nx (last updated on June 21, 2022).

## Phylogeographic Identification of >2 Virus Introduction Events

**Figure 1:**
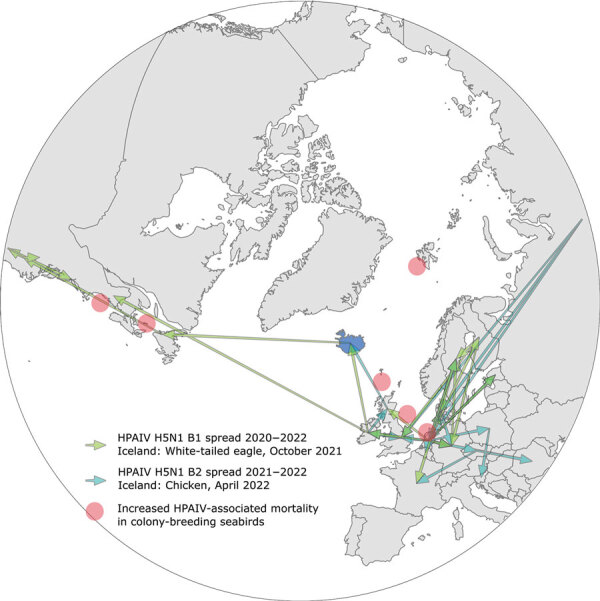
Polar map view of the palearctic and nearctic realm, and inferred spread of hemagglutinin clusters B1 and B2 of highly pathogenic avian influenza viruses (HPAIVs), subtype, clade 2.3.4.4b and their incursion routes to Iceland (blue) during 2021 (green arrows) and 2022 (turquoise arrows). Red dots indicate geographic locations where current (summer 2022) HPAIV-associated mass deaths in pelagic or colony-breeding seabirds have been reported. Data from were obtained from various sources (*15**–**18*).

We performed direct MinION (Oxford Nanopore Technologies, https://nanoporetech.com) full-genome sequencing as described (*5*) for 3 samples from Iceland (2022AI02104: white-tailed eagle, brain tissues; 2022AI02564 and 2022AI02565: backyard chickens, oropharyngeal swab specimens) that were immediately available for analysis and showed high viral loads. Presence of HPAIV H5N1 of clade 2.3.4.4b was confirmed. Phylogenetic and phylogeographic analyses of the genomes (Appendix) and associated data (*14*) showed close relationships to HPAIV H5N1 viruses from Europe and North America, grouping in 2 different HA clusters (B1 and B2) recently defined in clade 2.3.4.4b viruses from Europe (Figure 1) (*6**,**15**–**18*).

**Figure 2:**
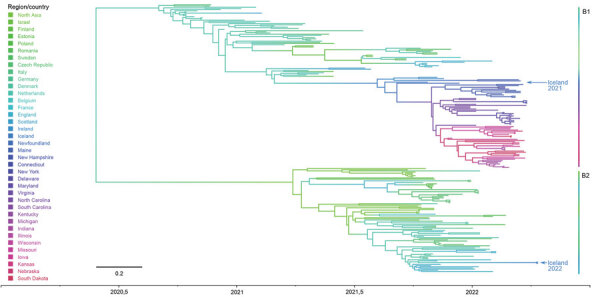
Phylogeographic tree of highly pathogenic avian influenza viruses. Taxa are colored according to their country of origin, and countries are arranged in geographic order from east to west. Arrows indicate viral genomes during 2021 and 2022 in Iceland and assigned to different hemagglutinin clusters B1 and B2. Method hints and basic data are presented in Hassan et al. (*13*). Scale bar indicates nucleotide substitutions per site.

Those findings point to >2 independent incursions into Iceland. The sequence from Iceland isolated during 2021 clusters in the B1 HA cluster between sequences from countries in northern Europe (the Netherlands, Ireland) and sequences from Canada and eastern coastal states of the United States (Figure 2). Analyses of concatenated genome sequences showed no evidence of reassortment with other AIV strains currently or recently circulating in Europe. Time-scaled phylogenetic analyses and inferred phylogeography (Figures 1, 2) demonstrate the circulation of similar viruses of the B1 HA cluster in northern Europe from the winter of 2020 to spring and summer of 2021 (*6*), and point toward viral spread from locations on the British Isles to Iceland and from there onwards to Canada and eastern coast of the United States.

White-tailed sea eagles are known to be a resident bird species in Iceland, and introduction of virus with this species is highly unlikely. Instead, the white-tailed sea eagle infection is likely caused by feeding of the eagle on infected, therefore weakened, prey or scavenging on carcasses, as described for raptor species (*19*). Some of the contemplable prey species of the taxonomic orders of *Anseriformes* or *Charadriiformes*, including geese, gulls and waders, are known to migrate from the British Isles and the North Sea region and are confirmed to have been infected in spring and early summer of 2021 in their overwintering areas (*20**–**22*). Iceland is situated along overlapping flyways that connect the Eastern and Western Hemispheres, and it has been suggested that Iceland connects virus movements between mainland Europe and North America (*7**–**11*,*23*).

In addition, HPAIV H5N1 genomes from 2 chickens dying in a backyard farm on Iceland during April 2022 were sequenced and could be traced back to a second, independent incursion featuring viruses of HA cluster B2. Inferred phylogeographic analysis showed that viruses collected in northern Asia were a possible source of this second introduction into central Europe and further spread throughout the continent (*6*). The Iceland chicken sequences cluster between viruses of HA cluster B2 collected from the British Islands and Ireland during the winter of 2021/2022 (Figures 1, 2). Viruses of this HA cluster (B2) have not been detected in North America to date.

## Epidemiologic, Conservational, and Public Health Concerns of Expanded HPAIV Circulation

Our data provide evidence for 2 translocation events of HPAIV H5N1 clade 2.3.4.4b viruses from central Europe through the British Isles into Iceland observed during October 2021 with a most recent ancestor in summer 2021 (most recent common ancestor 2021.5). Onward transmission to Newfoundland and possibly additional regions in the North Atlantic raises several concerns.

Large breeding colonies of pelagic bird species, such as puffins, northern gannets, and kittiwakes are located along the coasts of the North Atlantic. Confirmed HPAIV H5N1 infection in 9/12 gannet carcasses and daily public reporting of sick and dead gannets in the Reykjanes Peninsula, Iceland, since beginning of April 2022 underline that these colonies are now in danger of HPAIV H5 outbreaks of larger scale, which might affect the continuity of these local populations. Concerns extend to local populations of species with narrowly circumscribed breeding/resting ranges in the North Atlantic region such as great skua, long-tailed skua, red knots, pink-footed geese, and barnacle geese, as well as birds of prey exposed during opportunistic scavenging (e.g., white-tailed sea eagles and great skuas) and active hunting of weakened, infected prey (e.g., gyrfalcons [*Falco rusticolus*]). Therefore, enhanced passive surveillance should focus on such spots and scavenging and colony-breeding species.

The massively extended circulation in space and time of recent HPAIV H5N1 clade 2.3.4.4b viruses in migratory wild birds in the North Atlantic will further threaten endangered species. Grossly increased mortality rates for colonies of northern gannets and several tern species are being observed at several breeding sites throughout the North Atlantic (Figure 2). The most recent incursion of these viruses into wider palearctic areas of the Atlantic will inevitably lead to viral contamination of northern breeding habitats where ambient conditions prevail that are considered favorable for a prolonged retainment of viral infectivity outside avian hosts (*23**,**24*).

Increased alertness should now also extend to the Southern Hemisphere. In the 2 reported incursion events of gs/GD HPAI viruses into North America by migrating wild birds, during 2014 and 2021/2022, virus spread along the Pacific (2014) and the Atlantic coastline (2021) from north to south and further inland affecting wild birds and poultry in Canada, as well as in most of the United States (*7*–*9*). However, for unknown reasons, spread seems to be interrupted between North America and South America because no incursions had been reported during 2014/2015 or since 2021 from the Caribbean region and South America.

Similar observations have been made along the east side of the Pacific Ocean. Despite endemic presence of gs/GD HPAIV in several regions of Southeast Asia, and frequent incursions into migratory wild bird populations, cases have so far not been reported from Australia/Oceania (*4*). It is only at the most southern tip of Africa that gs/GD-like HPAIVs have reached and stayed within the Southern Hemisphere. However, this bridgehead of the virus might put geographically sequestrated subantarctic species, such as penguins and albatrosses, or the highly endangered avifauna of New Zealand at increased risk for exposure.

In conclusion, as shown by the rapid and devastating spread of HPAIV H5N1 through poultry holdings in North America after primary incursions from infected wild birds (*10*), the avian‒human interface has expanded again. Infections in 1 human (*25*) and in several terrestrial scavenging carnivores, such as foxes, skunks, and raccoons (*12*), illustrate the increased risk for spillover transmissions.

Appendix Additional information on Iceland as stepping stone for spread of high pathogenicity avian influenza between Europe and North America.
