# Supplementary material for: Iceland as Stepping Stone for Spread of Highly Pathogenic Avian Influenza Virus between Europe and North America
Source: Emerg Infect Dis. 2022 Dec;28(12):2383–8. doi: 10.3201/eid2812.221086 (PMC9707596; doi:10.3201/eid2812.221086)
Supplement: Appendix — Additional information on Iceland as stepping stone for spread of high pathogenicity avian influenza between Europe and North America. [file 22-1086-Techapp-s1.pdf]

Article DOI: https://doi.org/10.3201/eid2812.221086

Iceland as Stepping Stone for Spread of Highly Pathogenic Avian Influenza Virus between Europe and North America

Appendix

Methods

Full-genome sequencing of avian influenza (AIV)–positive samples was performed by using a previously described nanopore-based real-time sequencing method and previous full-genome amplification (*1*). RNA extraction with a Mini Viral Kit (QIAGEN, https://www.qiagen.com) and subsequent genome amplification with universal AIV-End-RT-PCR using Superscript III One-Step and Platinum Taq (ThermoFisher Scientific, https://www.thermofisher.com) with 1 primer pair (Pan-IVA-1F: 5′-TCCCAGTCACGACGTCGTAGCGAAAGCAGG-′3; Pan-IVA-1R: 5′-GGAAACAGCTATGACCATGAGTAGAAACAAGG-′3), binding to the conserved ends of the AIV genome segments, was conducted. After purification of the PCR products with AMPure XP Magnetic Beads (Beckman-Coulter, https://www.beckman.com), full-genome sequencing on a MinION Platform (Oxford Nanopore Technologies, https://nanoporetech.com) using the Rapid Barcoding Kit (SQK-RBK004; Oxford Nanopore Technologies) for transposon-based library preparation and multiplexing was performed.

Sequencing was directed according to the manufacturer’s instructions (Oxford Nanopore Technologies) with an R9.4.1 Flow Cell on an Mk1C Device with MinKNOW Software Core version 4.3.11 (Oxford Nanopore Technologies). Live base calling of the raw data with Guppy version 5.0.13 (Oxford Nanopore Technologies) was followed by a demultiplexing, quality check and trimming step to remove low quality, primer, and short (<50 bp) sequences. After sequencing, full-genome consensus sequences were generated in a map-to-reference approach using MiniMap2 (*2*). Reference genomes are a curated collection of all hemagglutinin and neuraminidase subtypes alongside an assortment of internal gene sequences chosen to cover all potentially circulating viral strains. Polishing of the final genome sequences was performed

manually after consensus production according to the highest quality (60%) in Geneious Prime version 2021.0.1 (Biomatters, https://www.geneious.com).

Segment specific and concatenated whole-genome multiple alignments were generated by using MAFFT version 7.450 (*3*), and subsequent maximum-likelihood trees were calculated by using RAxML version 8.2.11 (*4*) and model GTR GAMMA with rapid bootstrapping and search for the best scoring maximum-likelihood tree supported with 1,000 bootstrap replicates or alternatively with FastTree version 2.1.11 (*5*). Time-scaled trees of concatenated sequences of the different genotypes were calculated by using the BEAST version v1.10.4 software package (*6*) and a generalized time reversible plus gamma substitution model, an uncorrelated relaxed clock with a lognormal distribution, and coalescent constant population tree models. Chain lengths were set to 50 million iterations and convergence checked by using Tracer version 1.7.1 (https://beast.community). Time-scaled summary maximum clade credibility trees (MCCs) with 10% post burn-in posterior were created by using TreeAnnotator version 1.10.4 (https://beast.community) and visualized by using FigTree version 1.4.4 (http://tree.bio.ed.ac.uk). The MCC trees show 95% highest posterior density CIs at each node and posterior confidence values as branch support. Spatiotemporal spread was inferred on MCC trees with country traits by using SPREAD version 1.0.7 (*7*) and visualized with QGIS version 3.24.3 (QGIS.org). Real time RT-PCRs generically detecting the matrix gene segment of influenza A viruses, as well as subtype-specific and pathotype-specific quantitative reverse transcription PCRs have been performed as described (*8*).

References

1. King J, Harder T, Beer M, Pohlmann A. Rapid multiplex MinION nanopore sequencing workflow for influenza A viruses. BMC Infect Dis. 2020;20:648. PubMed https://doi.org/10.1186/s12879-020-05367-y

2. Li H. Minimap2: pairwise alignment for nucleotide sequences. Bioinformatics. 2018;34:3094–100. PubMed https://doi.org/10.1093/bioinformatics/bty191

3. Katoh K, Standley DM. MAFFT multiple sequence alignment software version 7: improvements in performance and usability. Mol Biol Evol. 2013;30:772–80. PubMed https://doi.org/10.1093/molbev/mst010

4. Stamatakis A. RAxML version 8: a tool for phylogenetic analysis and post-analysis of large phylogenies. Bioinformatics. 2014;30:1312–3. PubMed https://doi.org/10.1093/bioinformatics/btu033

5. Price MN, Dehal PS, Arkin AP. FastTree: computing large minimum evolution trees with profiles instead of a distance matrix. Mol Biol Evol. 2009;26:1641–50. PubMed https://doi.org/10.1093/molbev/msp077

6. Suchard MA, Lemey P, Baele G, Ayres DL, Drummond AJ, Rambaut A. Bayesian phylogenetic and phylodynamic data integration using BEAST 1.10. Virus Evol. 2018;4:vey016. PubMed https://doi.org/10.1093/ve/vey016

7. Bielejec F, Rambaut A, Suchard MA, Lemey P. SPREAD: spatial phylogenetic reconstruction of evolutionary dynamics. Bioinformatics. 2011;27:2910–2. PubMed https://doi.org/10.1093/bioinformatics/btr481

8. Hassan KE, Ahrens AK, Ali A, El-Kady MF, Hafez HM, Mettenleiter TC, et al. Improved subtyping of avian influenza viruses using an RT-qPCR-based low density array: 'Riems influenza A typing array', version 2 (RITA-2). Viruses. 2022;14:415. PubMed https://doi.org/10.3390/v14020415
